# Lithium Improves Hippocampal Neurogenesis, Neuropathology and Cognitive Functions in APP Mutant Mice

**DOI:** 10.1371/journal.pone.0014382

**Published:** 2010-12-20

**Authors:** Anna Fiorentini, Maria Cristina Rosi, Cristina Grossi, Ilaria Luccarini, Fiorella Casamenti

**Affiliations:** Department of Pharmacology, University of Florence, Florence, Italy; University of Missouri, United States of America

## Abstract

**Background:**

Alzheimer's disease (AD) is a neurodegenerative disorder characterized by progressive deterioration of cognitive functions, extracellular β-amyloid (Aβ) plaques and intracellular neurofibrillary tangles within neocortex and hippocampus. Adult hippocampal neurogenesis plays an important role in learning and memory processes and its abnormal regulation might account for cognitive impairments associated with AD.

**Methodology/Principal Findings:**

The double transgenic (Tg) CRND8 mice (overexpressing the Swedish and Indiana mutations in the human amyloid precursor protein), aged 2 and 6 months, were used to examine *in vivo* the effects of 5 weeks lithium treatment. BrdU labelling showed a decreased neurogenesis in the subgranular zone of Tg mice compared to non-Tg mice. The decrease of hippocampal neurogenesis was accompanied by behavioural deficits and worsened with age and pathology severity. The differentiation into neurons and maturation of the proliferating cells were also markedly impaired in the Tg mice. Lithium treatment to 2-month-old Tg mice significantly stimulated the proliferation and neuron fate specification of newborn cells and fully counteracted the transgene-induced impairments of cognitive functions. The drug, by the inhibition of GSK-3β and subsequent activation of Wnt/ß-catenin signalling promoted hippocampal neurogenesis. Finally, the data show that the lithium's ability to stimulate neurogenesis and cognitive functions was lost in the aged Tg mice, thus indicating that the lithium-induced facilitation of neurogenesis and cognitive functions declines as brain Aβ deposition and pathology increases.

**Conclusions:**

Lithium, when given on time, stimulates neurogenesis and counteracts AD-like pathology.

## Introduction

In the adult Central Nervous System (CNS), neurogenesis occurs throughout the lifetime. Adult neurogenesis is a process by which new neurons are produced from neural stem cells (NSCs) and has consistently been found in two “neurogenic regions” of the brain in vivo: the subgranular zone (SGZ) of dentate gyrus (DG) in the hippocampus, which supplies new neurons for the dentate granular cell layer (GCL) and the subventricular zone (SVZ) lining the lateral ventricles in the forebrain, which supplies new interneurons for the olfactory bulb [1],[2]. Adult NSCs have the ability to self-renew and to differentiate into neurons, astrocytes and oligodendrocytes in all mammalian CNS, including humans [3]. In both SGZ and SVZ neurogenic regions, neurogenesis progress as a multi-step process which starts with the proliferation of NSCs. For the hippocampus, conceptually, this process has been divided into four steps: (i) proliferation of NSCs, (ii) neuronal fate determination of NSCs, (iii) survival and maturation of new neurons and (iv) functional integration of new neurons into the pre-existent neuronal network [4]. Functionally, hippocampal neurogenesis appears to play an important role in learning and memory processes and mood regulation and its abnormal regulation might account for cognitive impairments associated with Alzheimer's disease (AD) and may underlie neuropsychiatric disorders like major depression [5]–[7]. Irregularities in adult neurogenesis in patients and A*β*PP overexpressing models of AD have been previously observed and reported. Some, although not all, in vivo studies using AD animal models indicate that decreased neurogenesis may be due to the increased deposition of amyloid-β (Aβ) and the subsequent formation of Aβ plaques [8] and in vitro studies suggest that proliferation of neuronal stem/progenitor cells is inhibited by Aβ peptide [9].

Stem cells differentiation is controlled by both intrinsic and extrinsic regulators. Among the extracellular factors that regulate this process are found the Wnt ligands. Of note, it has been shown that Wnt-3 is expressed near the SGZ in adult mice, that the Wnt canonical pathway plays an active role in the adult hippocampus [10] where it regulates adult neurogenesis promoting proliferation and differentiation of NSC (see ref. in [11]and in [12]). Glycogen synthase kinase-3β(GSK-3β) inhibitors stimulate neuronal differentiation and the mood stabilizer lithium, through activation of the canonical Wnt pathway, enhances proliferation of adult hippocampal progenitors *in vitro* inducing them to become neurons at therapeutically relevant concentrations [13].

In this study we sought to determine the effects of 5 weeks lithium treatment to TgCRND8 mice, of 2 and 6 months of age, representing the early and advanced stages of Aβ deposition respectively, on hippocampal neurogenesis and how it correlates with a reduced brain pathology and behavioural impairments. Here we demonstrate that lithium, via inhibition of GSK-3 and subsequent activation of Wnt/β-catenin signalling, stimulates adult hippocampal progenitor cells proliferation and neuronal differentiation, ameliorates cognitive functions and reduces Aβ deposition in 3-month-old TgCRND8 mice. The ability of lithium treatment to stimulate hippocampal neurogenesis and to ameliorate cognitive impairments is lost in advanced stage of the disease in the TgCRND8 mice.

## Results

### Cell proliferation in the SGZ of 3-month-old wild type and TgCRND8 mice and effect of lithium salts

**Figure 1:**
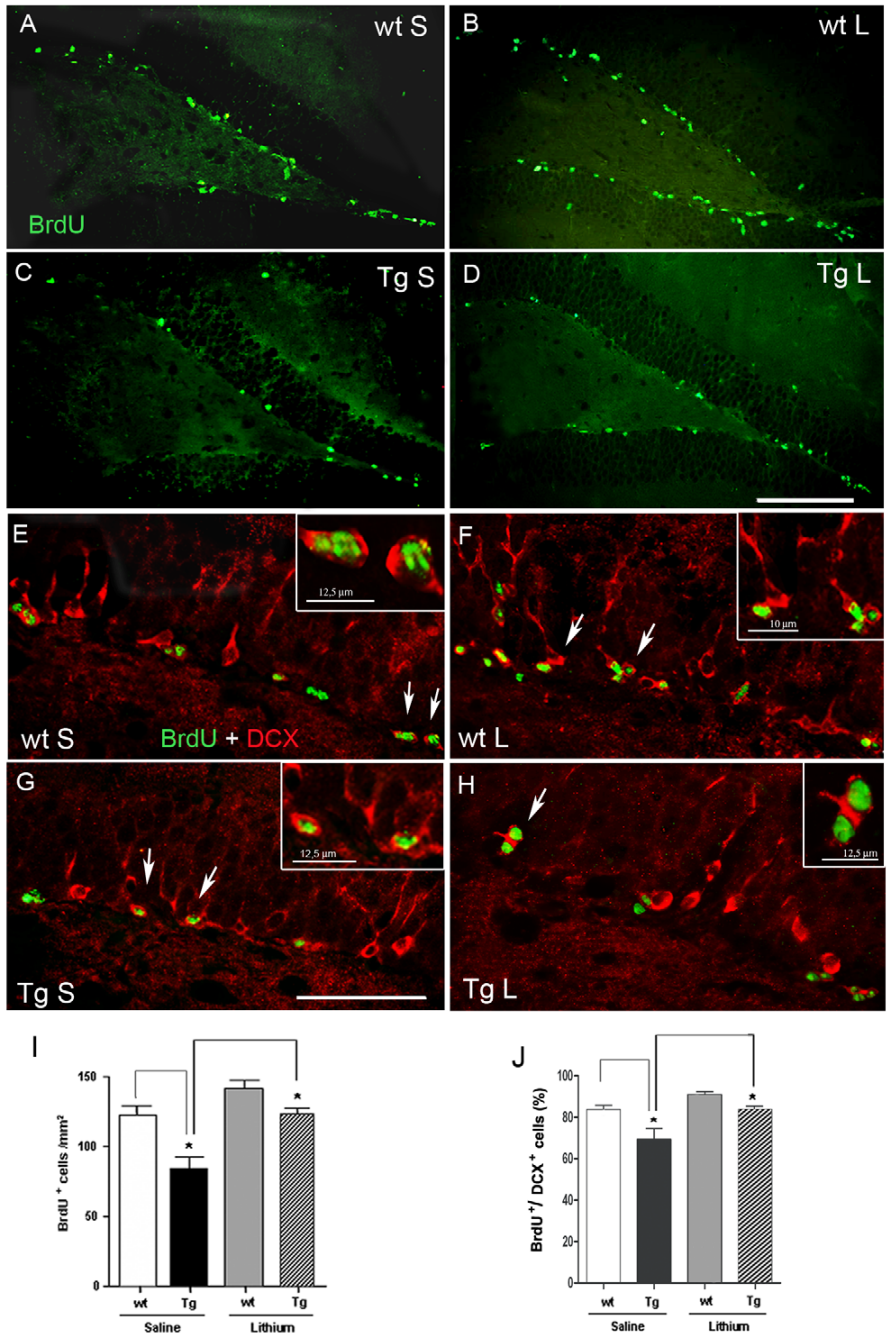
Cell proliferation in the SGZ of 3-month-old wt and TgCRND8 mice and effect of lithium treatment. A–D: BrdU^+^ (green) cells of neurogenic dentate gyrus area of saline-treated wt (A) and Tg (C) and of lithium-treated wt (B) and Tg (D) mice. E–H: double labelling immunohistochemical analysis for BrdU and DCX staining showing that the vast majority of BrdU^+^ cells (green) co-localized with DCX (red). White arrows indicate some co-localizing cells. Scale bars: 100µm (A–D), 50 µm (E–H). Insets in E–H: higher magnifications of the cells indicated by the arrows. I and J, quantitative analysis of the number of BrdU^+^ cells and of the new immature neurons (BrdU^+^/DCX^+^), respectively, in the SGZ of wt and Tg groups. The number of the BrdU^+^cells and the percentage of the BrdU^+^/DCX^+^ cells were significantly reduced in the saline-treated Tg compared to saline-treated wt mice: *P<0.05. Lithium significantly increased the number of these proliferating cells: *P<0.05, lithium-treated Tg vs saline-treated Tg mice. One-way ANOVA plus Bonferroni's post-comparison test. wt: wild type; Tg: TgCRND8, S: saline, L: lithium.

In the attempt to examine whether or not the Aβ deposition triggers progenitor cell proliferation, saline-injected control and Tg mice were administered BrdU during the last 3 days of treatment and were then sacrificed 24 h after the final BrdU injection. Mice of both genotypes displayed newly generated cells mostly in the SGZ of DG of the hippocampus, as shown by the fluorescent BrdU immunoreactivity (IR) (Fig. 1 A,C), which labels the nuclei of replicating cells irrespective of cell lineage. Notably, quantitative analysis of BrdU^+^ cells revealed a significant 31% reduction in the number of proliferating cells in the saline-treated Tg compared to saline-treated wt mice (Fig. 1 I), thus indicating that the neurodegenerative process associated with the transgene expression dramatically dampens the proliferation. Following lithium treatment the proliferation of neural progenitor cells in the SGZ of DG appeared to be increased in both wt and Tg mice (Fig. 1 B,D). Quantitative analysis of the fluorescent BrdU^+^ cells demonstrated a significantly higher number of proliferating cells in the lithium-treated Tg than in saline-treated Tg mice (Fig. 1 I). In order to determine whether these newly generated cells commit to neuronal differentiation, a double staining with DCX and BrdU antibodies was performed. At 24 h after BrdU administration the vast majority of BrdU^+^ cells in the SGZ was found within DCX^+^ cells in all groups (Fig. 1 E–H). Next, we counted and quantified the number of BrdU^+^/DCX^+^ stained cells and statistical analysis revealed a significant reduction in the percentage of newborn neurons in the saline-treated Tg compared to saline-treated wt mice (Fig. 1 J). In the Tg mice lithium treatment significantly increased the neuronal proliferation of BrdU^+^ cells up to control values (Fig. 1 J). Double labelling immuno-histochemistry with BrdU and GFAP antibodies revealed no or very few BrdU^+^/GFAP^+^ stained cells in the SGZ of both wt and Tg mice, regardless of treatment.

### Newborn cell survival and migration into the dentate gyrus

**Figure 2:**
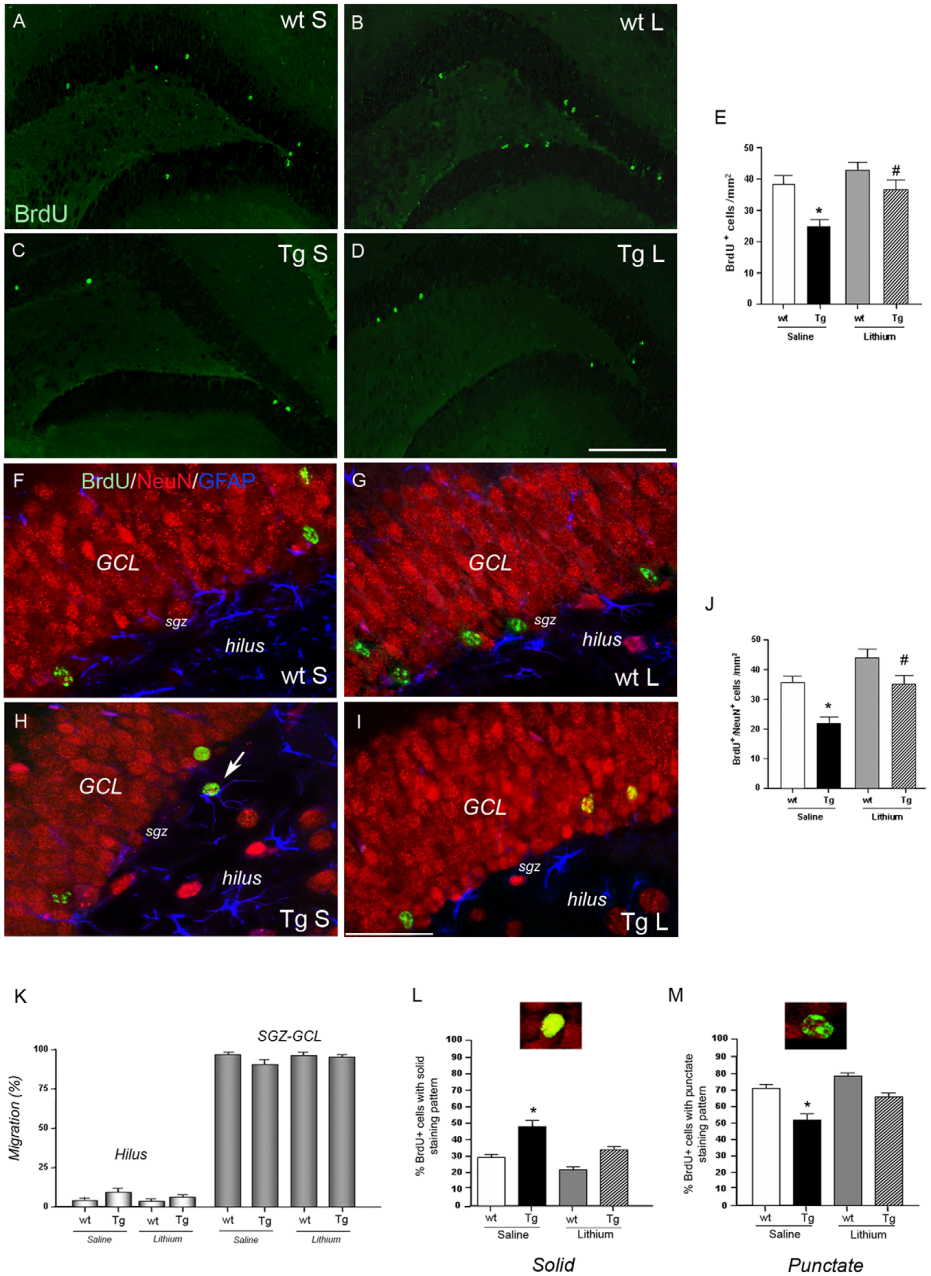
Cell survival in the SGZ-GCL of 3-month-old wt and TgCRND8 mice and effect of lithium treatment. A–D: immunohistochemical detection of BrdU^+^ cells (green) 5 weeks after the last administration of BrdU in the saline (A) and lithium (B)-treated wt and in the saline (C) and lithium (D)-treated Tg mice. Scale bar = 200 µm (A–D). E: quantification of the BrdU^+^ cells, *P<0.05, Tg vs wt, saline-treated group; ^#^P<0.01 lithium-treated Tg vs saline-treated Tg mice. F–I: triple labelling immunohistochemistry with BrdU (green), NeuN (red) and GFAP (blue) antibodies demonstrating the co-localization of BrdU and NeuN and their migration into the SGZ-GCL in the saline (F)- and lithium (G)-treated wt and saline (H)- and lithium (I)-treated Tg mice. Arrow in H indicates a newborn cell in the hilus region. Scale bar = 50 µm (F–I); J: quantitative analysis of the number of BrdU^+^ that was NeuN^+^ in the SGZ-GCL. A lower number of BrdU^+^/NeuN^+^ cells was found in the saline-treated Tg compared to saline-treated wt mice (*P<0.05) and this number recovered to control values following lithium treatment (^#^P<0.01, lithium-treated Tg vs saline-treated Tg mice). K: quantitative analysis of the number of newly-born neuron migrated into SGZ-GCL and hilus regions. L: quantitative analysis of the number of newborn cells with a solid BrdU appearance and a representative photomicrograph showing a cell with a solid staining pattern. *P<0.05, saline-treated Tg vs wt groups and vs lithium-treated Tg mice. M: quantitative analysis of the number of newborn cells with a punctate BrdU appearance and a representative photomicrograph showing a cell with a punctate staining pattern. *P<0.05, saline-treated Tg vs wt groups and vs lithium-treated Tg mice. One-way ANOVA plus Bonferroni's post-comparison test. wt: wild type, Tg: TgCRND8, S = saline, L = lithium.

To judge the relevance of the neurogenesis process, the survival and differentiation of SGZ progenitors were examined 5 weeks after the last BrdU administration to an additional group of 2-month-old wt and Tg mice. Both saline-treated wt and Tg mice showed fewer BrdU^+^ cells compared to the respective wt and Tg mice at 24 h after BrdU injection (30% and 26% of survival in the wt and Tg mice, respectively), indicating that many of SGZ progenitors do not survive to maturity. Significantly fewer BrdU^+^ cells were detected (Fig. 2 C) and counted (Fig. 2 E, *P<0.05) in the SGZ of saline-treated Tg relative to saline-treated wt mice(Fig. 2 A). Importantly, lithium treatment completely recovered the survival rate of progenitors in the Tg mice (Fig. 2 D and E, ^#^P<0.01). To investigate the phenotype of these survived cells, triple immunohistochemical analysis of BrdU with NeuN and GFAP was carried out. BrdU^+^ cells almost entirely differentiated into neurons (Fig. 2, J vs E), being the majority detected in the SGZ-GCL and a few in the hilus (Fig. 2 F–I). Consistent with the immunohistochemical analysis, more than 90% of the newborn BrdU^+^ cells migrated into the SGZ-GCL neuronal network and the remaining into the hilus, with no differences between groups (Fig. 2 K). Quantitative analysis of the NeuN-immunoreactive BrdU^+^ cells within the SGZ-GCL (Fig. 2 J) demonstrated that more than 95% of the newborn cells were mature neurons and that new neuron survival was significantly (*P<0.05) lower in the saline-treated Tg mice compared to saline controls. Lithium treatment significantly (^#^P<0.01) restored the new neuron survival in the Tg mice. According to previous studies a variety of morphological characteristics such as size, shape and staining pattern reflect maturity of new cells [14]–[16]. Therefore we checked for the morphological aspect of the BrdU/NeuN co-stained cells. In the SGZ-GCL of saline-treated Tg mice an equal amount of BrdU^+^ cells with solid and punctate staining patterns was detected (Fig. 2 L,M), indicating that roughly a 50% of newborn cells reached maturity. Notably, in the lithium-treated Tg mice the percentage of BrdU^+^ cells with punctate aspect raised up to 70–80%, not differing from the wt groups. Taken together these data indicate that lithium treatment counteracts the abnormal maturation of newborn neurons which is undergoing in the Tg mice.

### Cell proliferation in the SGZ of aged wild type and TgCRND8 mice and effect of lithium salts

**Figure 3:**
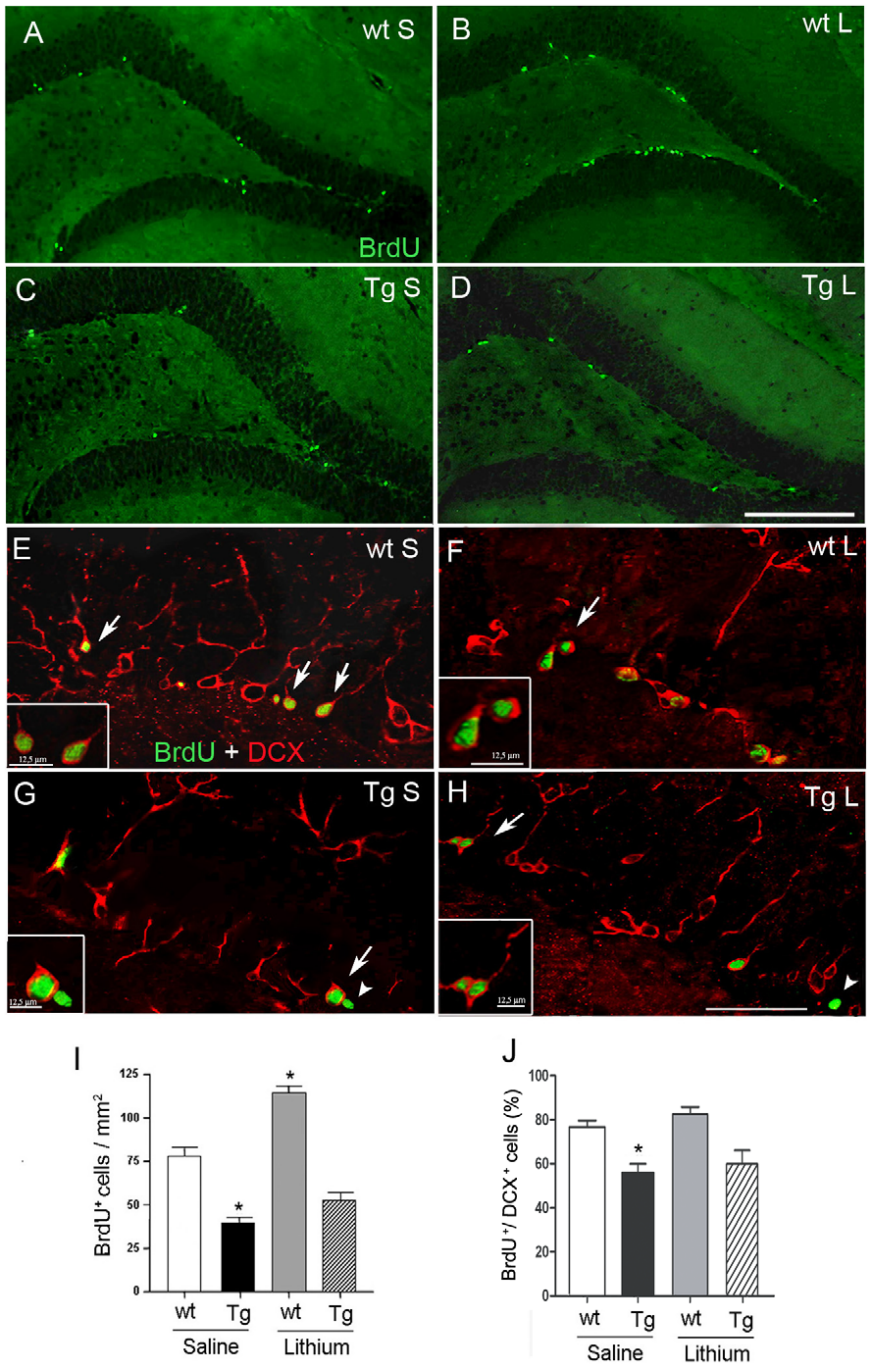
Cell proliferation in the SGZ of 7-month-old wt and TgCRND8 mice and effect of lithium salts. A–D: BrdU (green) immunohistochemical analysis of neurogenic dentate gyrus area in the hippocampus of saline-treated wt (A) and Tg (C) and of lithium-treated wt (B) and Tg (D) mice. Scale bar = 200 µm (A–D). E-H double labelling with BrdU (green) and DCX (red) in the SGZ of saline-treated wt (E) and Tg (G) and of lithium-treated wt (F) and Tg (H) mice. White arrows indicate co-localizing cells. White arrowheads in H and G indicate a non neuronal newborn cell. Scale bar = 50µm (E–H ). I: bar graph showing the number of BrdU^+^ cells within the neurogenic dentate gyrus area, *P<0.001 vs saline-treated wt mice. J: bar graph showing the percentage of BrdU^+^ cells that was DCX^+^ in the SGZ. *P<0.05, vs saline-treated wt mice. One-way ANOVA plus Bonferroni's post hoc test. wt: wild type; Tg: TgCRND8, S = Saline, L = Lithium.

In 7-month-old saline-treated TgCRND8 mice, characterized by an extensive cerebral Aβ deposition, plaque- associated gliosis and tau hyperphosphorylation [17], and in age-matched wt mice a few BrdU^+^ cells were detected in the SGZ (Fig. 3 A,C). In these mice the number of BrdU^+^ cells in the SGZ was significantly reduced compared to the respective 3-month-old mouse group (Tg mice: 39.5±1.96 in 7-month-old vs 84.27±8.38 in 3-month-old, −53%, P<0.01; wt mice: 77.06±3.49 in 7-month-old vs 122.59±6.76 in 3-month-old, −37%, P<0.01), indicating a decreased cell proliferation with age, irrespective of genotype. Moreover, in the 7-month-old saline-treated mice, quantitative analysis (Fig. 3 I) revealed a significant reduction in the number of BrdU^+^ cells in Tg compared to wt mice (−49%, *P<0.001). Altogether these findings indicate that cell proliferation is markedly reduced both by aging and the increasing severity of pathology in the transgenic mice. A 5-week lithium treatment to 7-month-old mice significantly increased the proliferation of BrdU^+^ cells in the wt mice, but not in the Tg mice, compared to saline treatment (^#^P<0.001). We next checked for the presence of BrdU/DCX co-labelled cells and found that the majority of newborn cells expressed DCX and were therefore committed to a neuronal phenotype, irrespective of both treatment and genotypes (Fig.3 E–H). Quantification of the percentage of BrdU^+^/DCX^+^ cells (Fig. 3 J) revealed a significant reduction in the saline-treated Tg mice compared to saline-treated wt mice (**P<0.05) and no enhancement in the percentage of newborn neurons was brought about by lithium treatment in both genotypes. As above reported for the group of 3 months, no or only a few BrdU^+^ cells differentiated into astrocytes, regardless of treatment.

### Effects of lithium salts on Aβ burden in the TgCRND8 mouse brain

**Figure 4:**
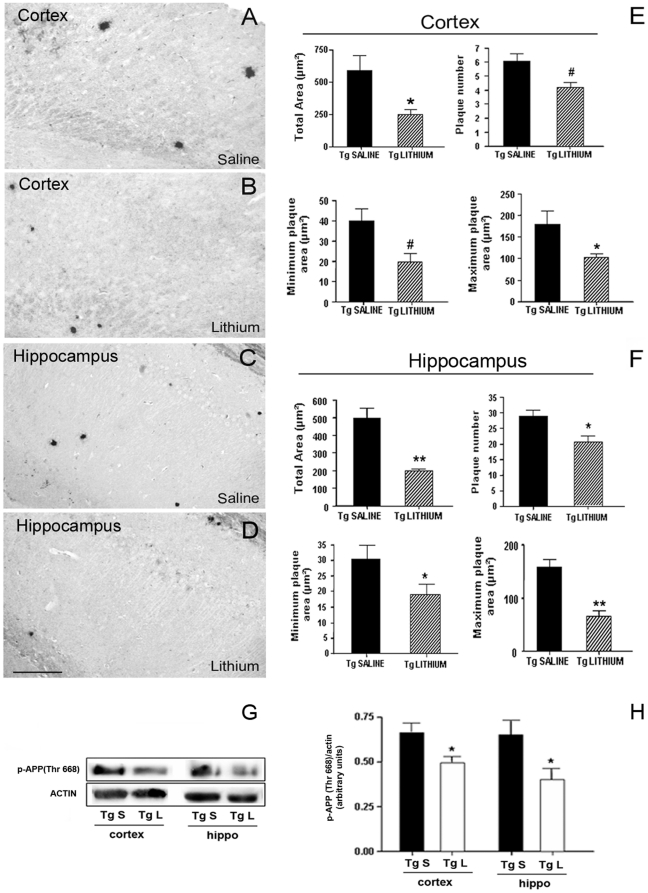
Lithium reduced Aβ burden and phospho-APP(Thr668) levels in 3-month-old TgCRND8 mouse brain. A–D representative photomicrographs of Aβ (1–42) immunolabelled amyloid plaques in the cortex (A,B) and hippocampus (C,D) of saline-treated (A,C) and lithium-treated (B,D) Tg mice. E,F: Quantitative analyses of the total area, plaque number and minimum and maximum plaque area in the cortex and hippocampus of saline-and lithium-treated Tg mice. Note in B and D and in the bar graphs the significant reduction of amyloid load by lithium treatment (Student's t-test, *P<0.05, ^#^P = 0.02, **P<0.01 versus Tg saline). Scale bar = 100 µm applies to all images. (G) qualitative Western blotting analysis of p-APP(Thr668) levels. β-actin was used as a protein loading control. (H) Quantitative analysis of protein levels showing a statistically significant decrease of p-APP(Thr668) levels in the cortex and hippocampus of lithium-treated Tg mice compared with saline-treated Tg controls. (One-way ANOVA plus Bonferroni's post hoc test,*P<0.05 versus Tg saline).

As next step we investigated whether the improved neurogenesis triggered by lithium treatment might result from a reduced amyloid load/plaques formation (Fig. 4 A–D). In the cortex and hippocampus of saline-treated 3-month-old Tg mice amyloid plaques were few and round shaped, ranging in size from small to medium, with a maximum plaque area of about 180 µm^2^. In these brain areas substantial differences in the amyloid load were brought about by 5 weeks of lithium treatment. Quantitative analysis of amyloid burden (Fig. 4 E,F) showed a significant lithium treatment effect in the total Aβ plaque area, in the plaque number and in the minimum and maximum plaque areas, both in the neocortex and hippocampus. Altogether, these findings demonstrate a robust lithium effect in the 3-month-old Tg mice with an early Aβ deposition. APP phosphorylated on threonine 688 (Thr688) by aberrant activation of GSK-3 has been reported to increase Aβ generation [18]. To investigate whether a reduction of phospho-APP(Thr668) was underlying the reduced Aβ burden the levels of phospho-APP(Thr668) were analyzed in 3-month-old TgCRND8 mice. By Western blotting, phospho-APP(Thr688) levels were significantly reduced in the cortex and hippocampus of lithium-treated Tg compared with saline-treated Tg mice (Fig. 4 G,H).

**Figure 5:**
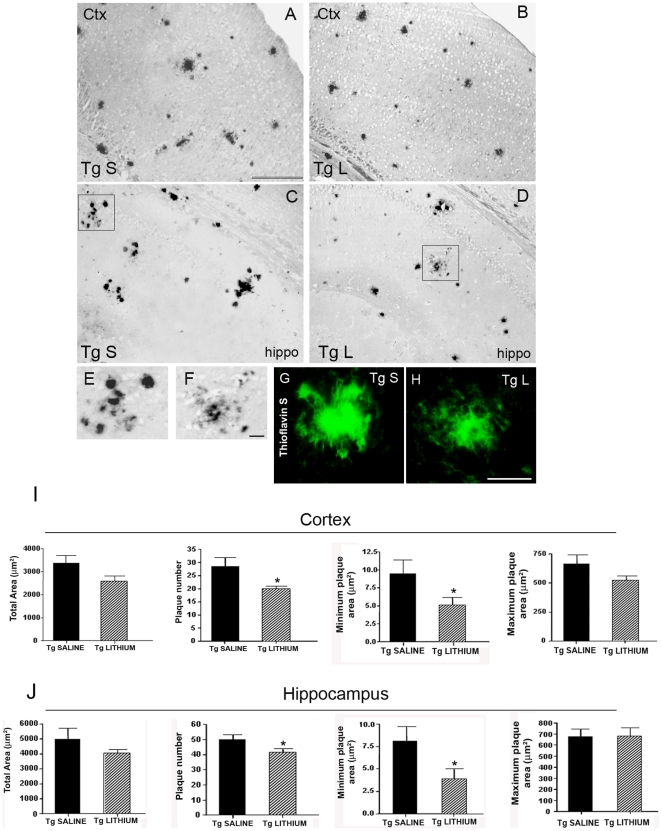
Lithium treatment reduced the de novo formation of Aβ plaques in 7-month-old TgCRND8 mouse brain. A–D: immunohistochemical detection of Aβ plaques in the cortex (A,B) and hippocampus (C,D) of saline-treated (A,C) and lithium-treated (B,D) 7-month-old TgCRND8 mice. Scale bar = 200 µm (A–D). E,F: higher magnifications of the squared areas in C and D, respectively. Note in F a radiating plaque in the lithium-treated group displaying a less dense core with fewer and smaller round-shaped deposits in its surroundings compared to the radiating plaque in E of the saline-treated group. Scale bar = 25 µm (E–F). G,H: Thioflavin S histochemistry of a radiating Aβ plaque in the saline (G)-and lithium (H)-treated Tg mice. Note in H the ribbon-like morphology of the β-amyloid plaque core and the reduction of compact deposits in the main amyloid core surroundings compared to the Aβ load in the G image. Scale bar = 25 µm (G–H). I,J: in bar graphs are reported the quantitative analyses of the total area, plaque number and minimum and maximum plaque area in the cortex and hippocampus of saline-and lithium-treated Tg mice. Only the plaque number and the minimum plaque area resulted significantly reduced by lithium treatment *P<0.05 versus Tg saline, Student's t-test.

In the cortex and hippocampus of 7-month-old saline-treated mice, amyloid plaques were numerous, ranging from small and round shaped, sometimes forming clusters, to big and radiating with a dense core and a maximum plaque area of about 700 µm^2^ (Fig. 5 A,C). A 5-week lithium treatment significantly reduced the plaque number and the minimum plaque area (Fig. 5 B,D). A more in depth morphological analysis revealed that in the lithium-treated group some radiating plaques displayed a less dense core with fewer and smaller round-shaped deposits in its surroundings compared to controls, as exemplified in the higher magnifications for the hippocampus (Fig. 5 E, F). Consistent with this, Thioflavin S histochemistry showed in the brain of lithium-treated 7-month-old Tg mice several radiating β-sheeted plaques with ribbon-like/diffuse cores and less compact deposits in their surroundings (Fig. 5 H). Taken together, these findings suggest that the lithium's ability to reduce the formation of Aβ deposits is maintained at the late stage of the pathology.

### Lithium salts reduced glia reaction in the brain of 3-month-old TgCRND8 mice

**Figure 6:**
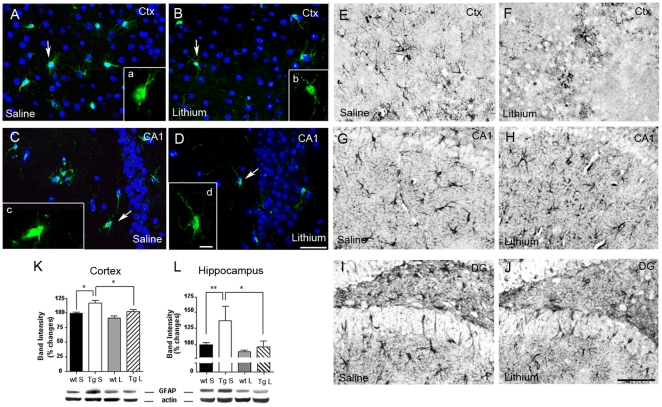
Lithium reduced glia activation in the 3-month-old TgCRND8 mouse brain. A–D: representative photomicrographs of merged fluorescent immunohistochemical analysis of IBA-1 (green) labelled microglial cells plus DAPI (blue) in the cortex (A,B) and CA1 area of the hippocampus (C,D) of saline-treated (A,C) and lithium-treated (B,D) TgCRND8 mice. Insets a-d: higher magnifications of the IBA-1 positive cells indicated by the arrows in each image. Note that microglial cells in the lithium treated mice have smaller cell bodies and thinner and elongated processes (magnified b and d images) than in saline controls, where microglial cells display swollen cell bodies and short and thick processes (magnified a and c images). Scale bar = 50 µm (A–D). Scale bar = 25 µm (a–d). E–J: representative photomicrographs of GFAP immunoreactivity in the cortex, CA1 and dentate gyrus of the hippocampus of saline- (E,G,I) and lithium- (F,H,J) treated TgCRND8 mice showing a reduced astrocytes reaction in the lithium group. Scale bar = 100 µm applies for all images. K,L: qualitative and quantitative analyses of GFAP Western blots in the cortex (K) and hippocampus (L) of saline- and lithium-treated Tg mice. Cortex: *P<0.05, saline-treated Tg vs saline-treated wt and vs lithium-treated Tg mice; hippocampus: **P<0.01 saline-treated Tg vs saline-treated wt mice, *P<0.05 saline-treated Tg vs lithium-treated Tg mice, one-way ANOVA plus Bonferroni's post hoc test. Ctx: cortex; DG: dentate gyrus.

Fluorescent immunohistochemical analysis revealed diminished parenchyma and plaque-associated inflammation in lithium-treated 3-month-old Tg mice, involving both IBA-1-labelled microglial cells and GFAP-immunoreactive astrocytes. IBA-1 antibody showed microglial cells with small cell bodies and thin and elongated processes, both in the neocortex and hippocampus, in the lithium-treated Tg (Fig. 6 B,D), compared to saline-treated Tg mice (Fig. 6 A,C), where microglial cells with enlarged cell bodies and short and thickened processes were found. By GFAP immunostaining hypertrophic astrocytes with long and thick branches were detected in the neocortex and in all the subregions of the hippocampus of saline-treated Tg mice (Fig. 6 E,G,I). By contrast, in the same brain areas of lithium-treated Tg mice (Fig. 6 F,H,J) astrocytes reaction occurred at much less extent. Accordingly, quantitative analysis of GFAP blots revealed a significant reduction of GFAP protein levels in the lithium-treated Tg mice compared to Tg controls (Fig. 6 K,L).

### Lithium salts inhibited GSK-3β in the TgCRND8 mouse brain

**Figure 7:**
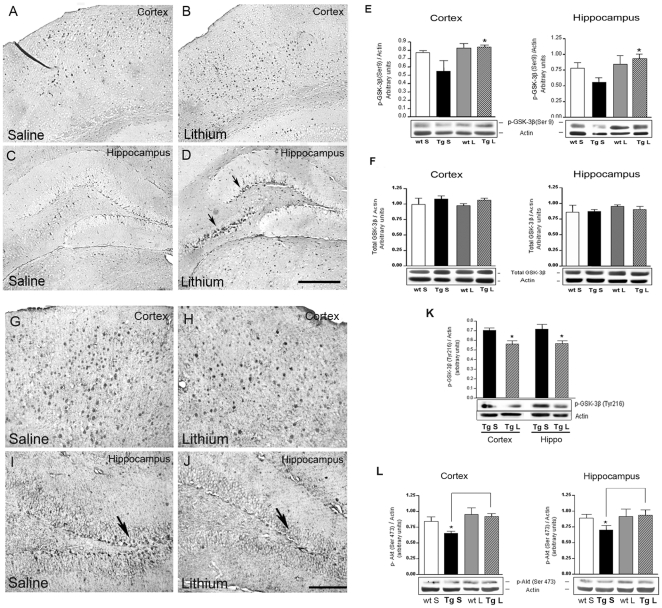
Effect of lithium treatment on the expression of GSK-3β isoforms and pAkt(Ser473) in the 3-month-old TgCRND8 mouse brain. A–D: representative photomicrographs of p-GSK-3β(Ser9) immunoreactivity in the cortex and hippocampus of saline (A,C)- and lithium (B,D)-treated TgCRND8 mice showing an increased p-GSK-3β(Ser9) immunoreactivity in the deep layers of the parietal neocortex (B) and in the hippocampus (D) of the lithium group compared to the saline group. Arrows in D indicate the p-GSK-3β(Ser9) immunoreactivity in the CA3 and dentate gyrus of the hippocampus. Scale bar: 100 µm (A–D). E and F: qualitative Western blotting and densitometric analysis of p-GSK-3β(Ser9) and total GSK-3β bands, respectively. β-actin serves as a protein loading control. Statistics indicate a significant increase of p-GSK-3β (Ser9) levels in the neocortex and hippocampus of lithium-treated 3-month-old Tg mice compared with the age-matched controls and no differences in the total GSK-3β expression among the groups. *P<0.05, vs saline-treated Tg. Two-way ANOVA plus Bonferroni's post hoc test. G–J: representative photomicrographs of active GSK-3β immunoreactivity in the cortex and dentate gyrus of the hippocampus of saline (G,I)- and lithium (H,J)-treated TgCRND8 mice. Note fewer active GSK-3β positive cells in the parietal neocortex (H) and hippocampus (J) of the lithium group compared to the saline group. Arrows in I and J indicate the GSK-3β immunoreactivity in the dentate gyrus. Scale bar:100 µm (G–J). K: qualitative Western blotting and densitometric analysis of p-GSK-3β(Tyr216) protein levels in the cortex and hippocampus of saline- and lithium-treated TgCRND8 mice. Statistics indicate a significant reduction of p-GSK-3β(Tyr216) levels in the cortex and hippocampus of lithium-treated 3-month-old Tg mice compared with the age-matched controls. Student's t test: *P<0.05, lithium-treated Tg vs saline-treated Tg mice. (L) qualitative Western blotting and densitometric analysis of pAkt(Ser473) in the cortex and hippocampus of saline- and lithium-treated wt and Tg mice. Statistics indicate a slight but significant increase in the levels of this kinase by lithium treatment in the Tg mice. Two-way ANOVA plus Bonferroni's post hoc test: *P<0.05, saline-treated Tg vs lithium-treated Tg mice. Data are presented as mean ± S.E.M. β-actin serves as a protein loading control. Ctx: Cortex; Hippo: hippocampus, S = saline, L = lithium, Tg: TgCRND8.

In neuronal cells lithium can inhibit GSK-3β either directly or indirectly by activating Akt, thus leading to increased Ser9 phosphorylation and consequent inactivation of the kinase. Wexler et al. [13] reported that the inhibition of GSK-3β by lithium results in the activation of the canonical Wnt signalling pathway which positively regulates adult neural progenitors. In order to examine whether lithium's ability to enhance neuronal differentiation was mediated by GSK-3β inhibition coronal brain sections from 3-month-old Tg mice were immuno-labelled with p-GSK-3β(Ser9) antibody, which is specific for the inactive isoform of the kinase. An increased immunoreactivity was found in the deep layers of the parietal neocortex and in the subfields of the hippocampus, as shown for the SGZ of DG and CA3 area, of the lithium-treated 3-month-old TgCRND8 mice as compared to the corresponding brain areas of saline-treated age-matched Tg mice (Fig. 7 A–D). Western blotting analysis detected higher p-GSK-3β(Ser9) protein levels in the entire hemicortex and hippocampus of lithium-treated Tg compared to saline-treated Tg mice (Fig. 7 E). Of note, no differences were found in the cortical and hippocampal levels of total GSK-3β among the four groups (Fig. 7 F). Accordingly, the expression of the active isoform of GSK-3β was significantly reduced, both in immunohistochemical and Western blotting analyses, in the cortex and hippocampus of lithium-treated 3-month-old TgCRND8 mice compared to saline-treated age-matched Tg controls (Fig. 7 G–K). In parallel, Western blotting experiments aimed at investigating Akt activation following lithium treatment were carried out employing p-Akt(Ser473) antibody, which is specifically raised against the activated isoform of the kinase. Quantification of protein band intensities demonstrated significant increases in p-Akt(Ser473) levels in the cortex and hippocampus of lithium-treated 3-month-old Tg mice as compared to the age-matched Tg controls (Fig. 7 L), indicating that an Akt-mediated inactivation of GSK-3β is also undergoing in lithium-treated animals.

**Figure 8:**
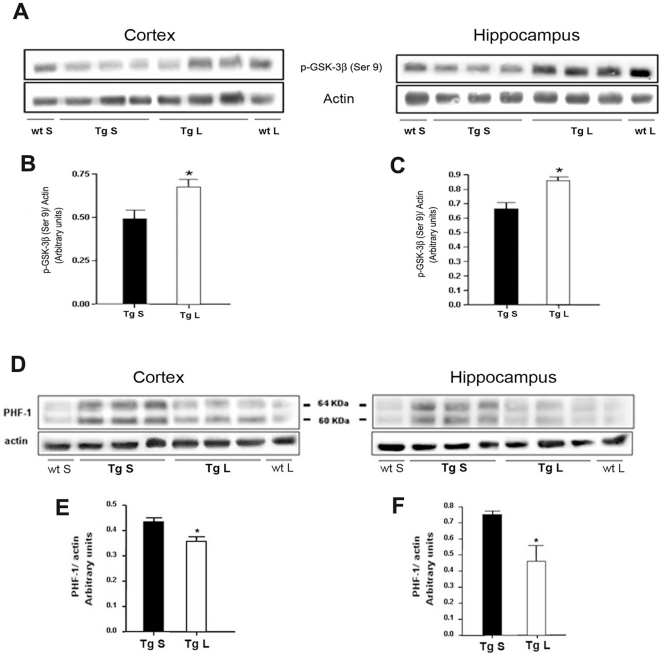
Effect of lithium treatment on the inactive p-GSK-3β(Ser9) and PHF-1 immunoreactivity in the 7-month-old TgCRND8 mouse brain. A: qualitative Western blotting and (B,C) densitometric analysis of p-GSK-3β(Ser9) in the cortex and hippocampus of 7-month-old TgCRND8 mice. Statistics indicate a significant increase of p-GSK-3β(Ser9) levels in the neocortex and hippocampus of lithium-treated 7-month-old Tg mice compared with the age-matched controls. *P<0.05, vs saline-treated Tg. Two-way ANOVA plus Bonferroni's post hoc. D: qualitative Western blotting and (E–F) densitometric analysis of PHF-1 protein levels in the cortex and hippocampus of saline- and lithium-treated Tg mice. Statistics indicate a significant reduction of PHF-1 levels in the neocortex and hippocampus of lithium-treated 7-month-old Tg mice compared with the age-matched Tg controls. Data are presented as mean ± S.E.M. *P<0.05, saline-treated Tg vs lithium-treated Tg mice, Student's t-test. β-actin serves as a protein loading control. S = saline, L = lithium, Tg: TgCRND8.

The inhibition of GSK-3β by lithium was achieved also in the group of 7-month-old TgCRND8 mice as demonstrated by the higher p-GSK-3β(Ser9) protein levels detected in the neocortex and hippocampus of lithium-treated Tg compared to saline-treated age-matched Tg mice (Fig. 8 A–C). In these aged mice we have previously reported the occurrence of abnormal tau hyperphosphorylation at different sites [17]. A large body of evidence indicates that GSK-3 activation, together with other tau kinases, plays a key role in tau phosphorylation, whereas its inhibition is neuroprotective [19], [20]. By Western Blotting we demonstrated a statistically significant reduction in the levels of PHF-1 immunopositive tau, both in the cortex and hippocampus of lithium-treated TgCRND8 mice as compared to saline-treated age-matched Tg mice (Fig. 8 D–F).

### Lithium salts activated canonical Wnt pathway in 3-month-old TgCRND8 mice

**Figure 9:**
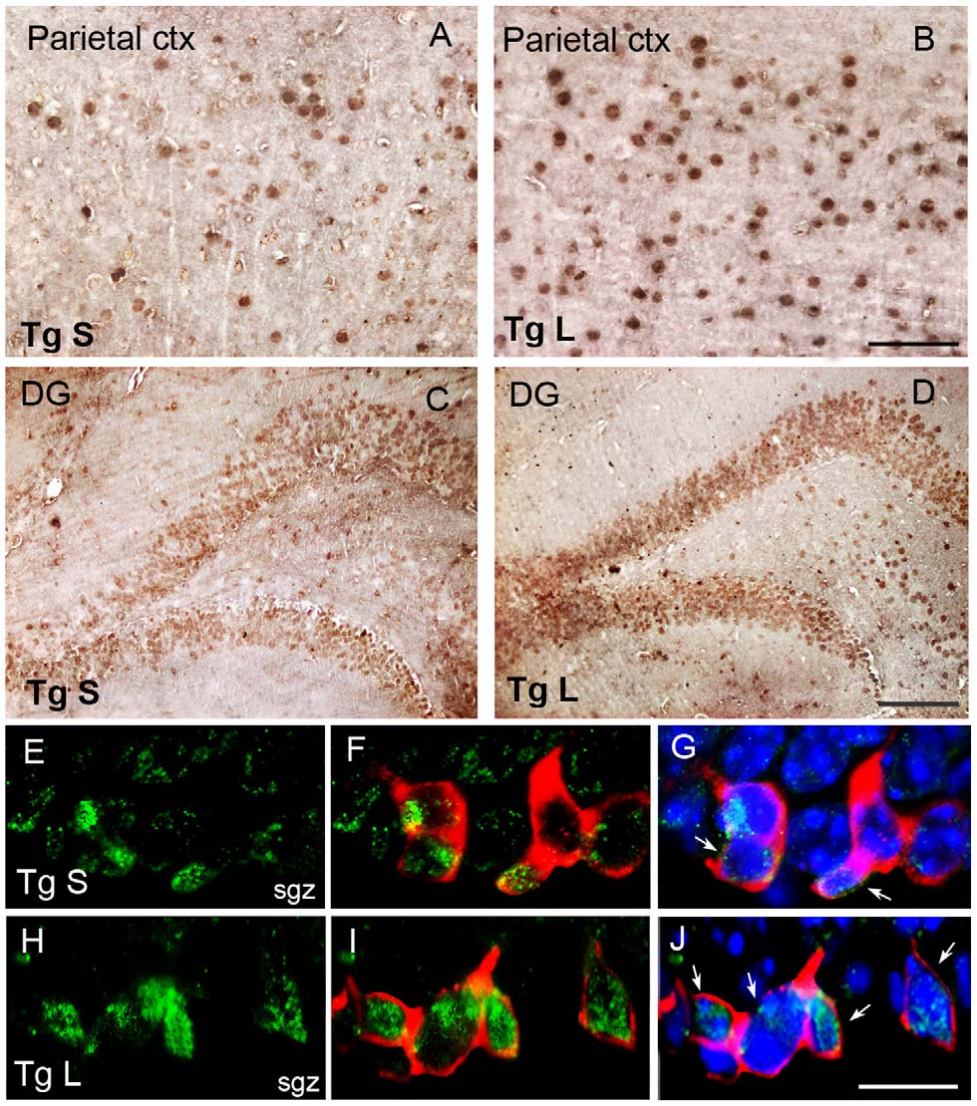
Lithium activated Wnt/β -catenin pathway in the SGZ of 3-month-old TgCRND8 mouse brain. A–D: single β-catenin immunohistochemistry in the saline (A,C)- and lithium (B,D)-treated Tg mouse brain. An apparently higher number of β-catenin positive cells is present in the parietal cortex and dentate gyrus of the hippocampus of lithium-treated Tg mice compared to Tg controls. Scale bar = 50µm (A–B). Scale bar = 100µm (C–D). E–J: triple labelling fluorescence for β-catenin (green), DCX (red) and DAPI (blue) staining. E–G: saline treated Tg mice, G merge of E and F images plus DAPI. H–J lithium-treated Tg mice, J merge of H and I images plus DAPI. White arrows in G and J indicate the nuclear co-localization of DCX and β-catenin staining in the saline-and lithium-treated Tg mice, respectively. Scale bar = 12.5 µm (E–J). sgz: sub granular zone, ctx: cortex; DG: dentate gyrus, Tg: TgCRND8, S = saline, L = lithium.

To determine the relative involvement of the canonical Wnt/β-catenin pathway activation in the lithium-induced neuronal proliferation and differentiation of hippocampal progenitors, downstream to GSK-3β, we evaluated, firstly, whether, as a consequence of GSK-3β inactivation, the intracellular expression of β-catenin was enhanced and, secondly, whether the new generated neurons expressed β-catenin. Single immunohistochemistry revealed increased β-catenin immunoreactivity throughout the neocortex and the subfields of the hippocampus, as shown for the GCL of DG, of lithium-treated Tg as compared to saline-treated Tg mice (Fig. 9 A–D). Next, in triple labelling fluorescence performed with DCX and β-catenin antibodies plus DAPI (Fig. 9 E–J), we observed that in the SGZ of lithium-treated Tg mice the number of DCX positive neurons (red) expressing β-catenin (green) at the nuclear level (Fig. 9 H–J) was higher than in the saline-treated Tg mice (Fig. 9 E–G). This suggests that lithium, by GSK-3β inhibition, facilitates β-catenin nuclear translocation and, thereby, promotes adult hippocampal neurogenesis *in vivo* by activating downstream Wnt/β-catenin target genes.

### Lithium salts improved cognitive impairments in 3-month-old but not in 7-month-old TgCRN8 mice

**Figure 10:**
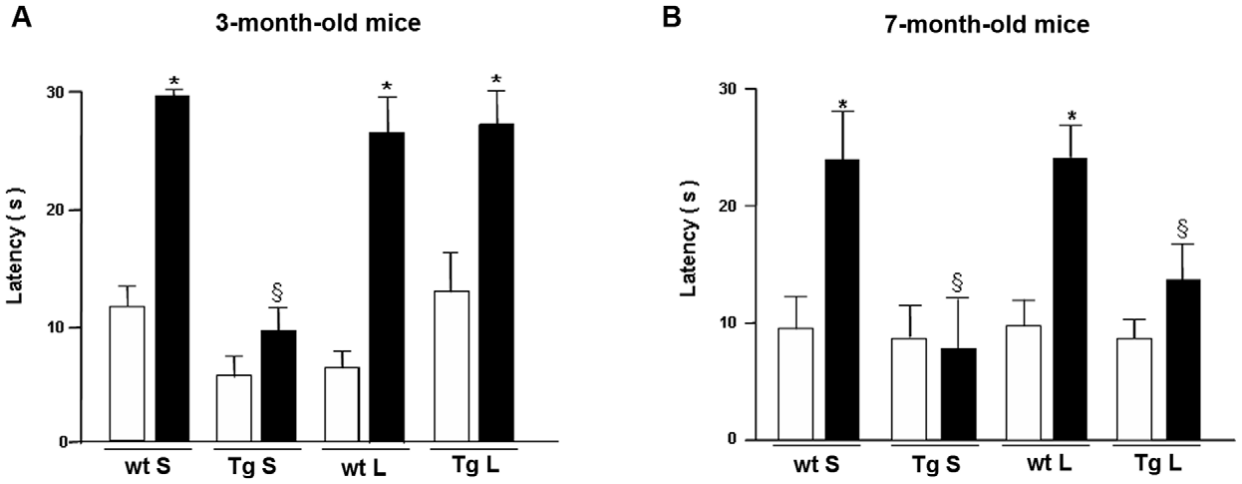
“Step-down” inhibitory avoidance task in saline- and lithium-treated 3- and 7-month-old wild type and TgCRND8 mice. No statistically significant differences are present in the mean training latencies among groups. In the group of 3 months of age, one-way ANOVA plus Bonferroni's post comparison test shows a statistically significant increase in the mean retention latencies in saline- and lithium-treated wt and lithium-treated Tg mice, as compared to their respective training latencies, *P<0.001. Saline-treated Tg mice do not show significant differences between training and retention latencies (P>0.05). The retention latencies of saline-treated Tg mice significantly differ from the retention latencies of all the other groups (§P<0.001). In the group of 7 months of age, one-way ANOVA plus Bonferroni's post comparison test shows a statistically significant increase in the mean retention latencies in saline-and lithium-treated wt mice, as compared to their respective training latencies, *P<0.05. No significant differences are revealed between training and retention latencies in saline-and lithium- treated Tg mice (P>0.05). The retention latencies of saline-and lithium-treated Tg mice are significantly different from the retention latencies of all other groups (§P<0.05). Data are reported as mean ± S.E.M. White bars = training latencies, black bars = retention latencies, S = saline, L = lithium, Tg: TgCRND8, wt: wild type.

At the end of the fourth week of treatment the mice were evaluated for cognitive functions in the “Step down” inhibitory avoidance test. During the training test no significant differences were observed between the four groups of animals of 3 and 7 months of age. In the 24 h retention test, the step-down latencies recorded for the saline-treated TgCRND8 mice were significantly reduced as compared to saline-treated wt mice. Lithium treatment resulted in a significant memory improvement of 3-month-old Tg mice (Fig. 10 A). These results indicate that lithium salts treatment to 2-month-old Tg mice is capable to completely prevent cognitive impairment. By contrast, lithium was unable to counteract the transgene-related cognitive impairments in 7-month-old Tg mice (Fig. 10 B).

**Figure 11:**
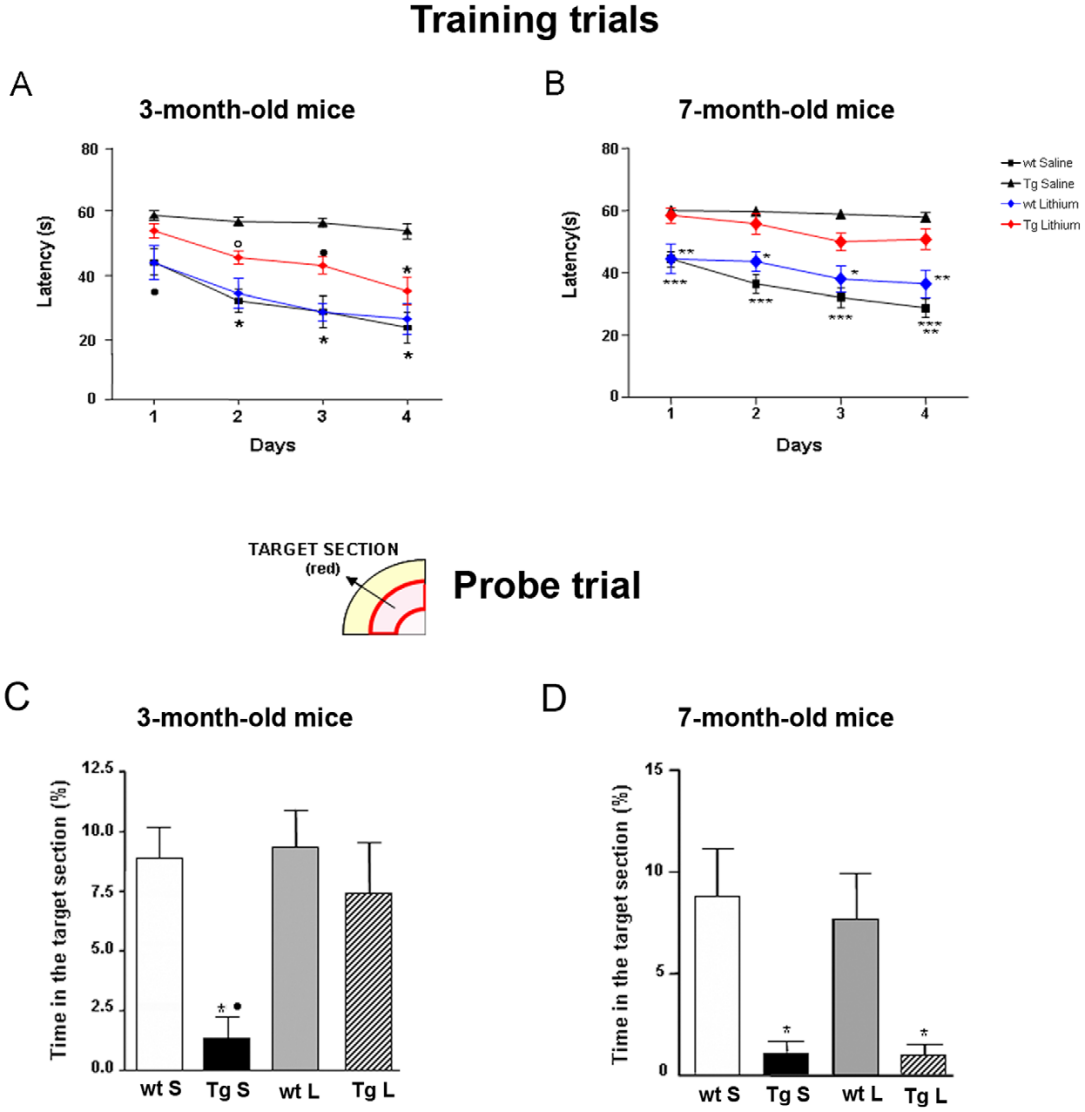
Effects of chronic lithium treatment on Morris water maze performances of wild type and TgCRND8 mice. A,B: training trials. Latencies to find the platform during the training session are reported as mean ± S.E.M. Each point represents the mean daily values of four trials per day. Statistics were performed using one-way ANOVA plus Bonferroni's post comparison test. In the group of 3 months of age, the saline- and lithium-treated wt mice show significantly shorter escape latencies with respect to saline-treated Tg mice over sessions (day 1: ^•^P<0.05, days 2–4: *P<0.001). In lithium-treated Tg mice the recorded latencies are significantly higher than those of saline-treated Tg mice (day 2: °P<0.05; day 3: ^•^P<0.05 and day 4: *P<0.001) and of saline- and lithium-treated wt mice (day 1: ^•^P<0.05). In the group of 7 months of age, saline-and lithium-treated wt mice perform at significantly shorter latencies as compared to the Tg groups over sessions (***P<0.001 saline-treated wt vs saline-treated Tg; **P<0.01 saline- and lithium-treated wt vs lithium-treated Tg mice *P<0.05 lithium-treated wt vs lithium-treated Tg). C, D: bar graphs showing the percentage of time spent in the target section during the probe trial within 30 s. Data are reported as mean ± S.E.M. Statistics were performed using one-way ANOVA plus Bonferroni's post comparison test. In the group of 3 months of age, saline-treated Tg mice spend a significantly shorter percentage of time in the target section than saline- and lithium-treated wt mice (*P<0.01) and lithium-treated-Tg mice (^•^P<0.05). In the group of 7 months of age, saline- and lithium-treated Tg mice are significantly impaired as compared to wt groups (*P<0.01). Top left is depicted the target section of the Morris water maze between red lines.

During the fifth week of treatment 3-month-old Tg and wt mice were trained for four days in the MWM task to learn where the hidden platform was located. The animals were naive to the water maze and showed no deficiencies in swimming abilities, directional swimming toward the platform, or climbing onto an hidden platform during training trials. Saline- and lithium-treated wt mice significantly shortened the escape latency during the acquisition phase over sessions (Fig. 11 A), they were good swimmers and responded for being placed in water with an appropriate swim-search response. Lithium-treated TgCRND8 mice showed reduced escape latency as compared to saline-treated Tg mice during the last three days of training. Two-way ANOVA revealed a significant lithium treatment effect on escape latency in Tg mice (F _(1,9)_ = 7,61, P<0.0001, treatment factor) and Bonferroni's post hoc multiple comparison test revealed significantly shorter escape latencies in the wt groups throughout the training period (P<0.001 saline- and lithium-treated wt vs saline-treated Tg) and in lithium-treated Tg on day 2 (°P<0.01), 3 (**^•^**P<0.05) and 4 (*P<0.001) with respect to saline-treated Tg mice, indicating that lithium markedly ameliorated cognitive abilities in Tg mice.

The development of spatial memory for the platform location during the training sessions was evaluated in a probe trial administered 5 h after the last test on day 4. The analysis revealed that lithium-treated Tg and saline- and lithium-treated wt mice spent a significantly higher percentage of time in the target section in which the platform was previously located than saline-treated Tg mice (*P<0.05, lithium-treated Tg vs saline-treated Tg; ^•^P<0.001 saline- and lithium-treated wt vs saline-treated Tg) (Fig. 11 C). The swim speed in quadrants was comparable among the groups, indicating that the mice were not impaired in motility and exploratory behavior. As above reported for the “step down” test, the performance of 7-month-old saline-treated Tg mice in the MWM was impaired as compared to age-matched saline-treated wt mice and lithium treatment was unable to produce any significant amelioration in all the MWM paradigms evaluated (Fig. 11 B,D).

## Discussion

The purpose of the present study was to examine whether chronic lithium treatment can potentially be effective in ameliorating the cognitive deficits associated with AD by stimulation of cell proliferation and survival and integration of newborn cells into neuronal network of the SGZ-GCL of the hippocampus in a mouse model of AβPP deposition. In addition, we sought to determine whether the facilitation of neurogenesis by lithium was through the activation of the Wnt/β-catenin signalling pathway. To this purpose we used TgCRND8 mice, harbouring two mutant human APP genes [21], at 3 and 7 months of age, representing the early and advanced stages of Aβ deposition. In this transgenic mouse strain, in agreement with findings in other mouse models of AD with distinct genetic mutations that underlie their pathology [22], [23] we have found a decreased neurogenesis in the SGZ of the hippocampus. The decrease of hippocampal neurogenesis was associated with behavioural deficits and developed with age, in parallel with the development of AD-like pathology presently and previously reported in this Tg mouse strain [17], [21], [24], [25]. The survival, differentiation into neurons and maturation of the proliferating cells were also markedly impaired in the transgenic mice. Lithium salts treatment to 2-month-old Tg mice significantly stimulated the proliferation and neuron fate specification of newborn cells and fully counteracted the transgene-induced impairments of cognitive functions. Moreover, our results showing lithium-induced GSK-3β inhibition and β-catenin increase, indicate that lithium promotes hippocampal neurogenesis through the activation of GSK-3β/β-catenin pathway and thereby enhances the expression of proneural genes. On the other side, our findings demonstrate that lithium's ability to stimulate neurogenesis and cognitive functions is impaired in the aged Tg mice characterized by an extensive Aβ plaques deposition and suggest that the pathway(s)/mechanism(s) underlying the lithium-induced facilitation of neurogenesis and cognitive functions decline as brain Aβ deposition and pathology increases.

Several mouse models recapitulating features of AD pathology have been used to investigate whether the process of neurogenesis is altered in the disease and whether it contributes to cognitive impairments (see refs in [26]). In *vitro* and *in vivo* studies reported a suppressive toxic effect of Aβ 1–42 on human and mouse neural stem cells [9]. Accordingly, we found that the number of proliferating cells in the SGZ undergoes a marked and progressive age- and Aβ load-related reduction. In the TgCRND8 mice the first Aβ deposits appear at the age of 3 months and, become numerous and widespread throughout the hippocampus and cortex at the older age of 7 months [21]. Not only the proliferation of neural progenitor cells was decreased in the Tg mice, but also their differentiation into mature neurons and functional integration into the pre-existing neuronal network were more strongly reduced in the Tg mice compared to controls. Newborn neurons leaving the SGZ have been shown to migrate mainly into the adjacent GCL of the DG of adult rodents [27]. Accordingly, 5 weeks after the last BrdU administration we found that the majority of BrdU^+^ cells migrated into the adjacent granular layer and a very few cells into the hilus, irrespective of genotype. Newborn cells, influenced by multiple factors, undergo complex stages of morphological and functional maturation [28]. After a 5-week survival period, the newborn cells in the SGZ-GCL displayed either solid or punctate BrdU staining, punctate BrdU positive neurons were significantly lower in the TgCRND8 mice compared to controls, likely as a consequence of the hippocampal Aβ deposition as well as other age-related dysfunctions. The reduction of newborn cells with a punctate BrdU staining in the Tg mice likely reflect a retarded maturation in newborn SGZ-GCL neurons, as previously reported [14], [29].

The newly generated neurons in the hippocampal formation and their incorporation into DG circuits support spatial memory (for refs see [30]) and cognitive stimuli promote the survival of newborn cells [31]. Thus, an interplay seems to exist in the hippocampus between neurogenesis and learning and memory functions which in the young TgCRND8 mice is positively modulated by lithium. In fact, lithium administration for 5 weeks to 2 month-old Tg mice significantly increased cell proliferation, neuronal differentiation, survival and correct maturation of BrdU^+^ cells in the SGZ-GCL and significantly ameliorated the behavioural performance in the MWM and “Step down” tests. At the same time the entire Aβ burden was significantly reduced, likely as a prevention of Aβ deposition, by 5 weeks of lithium treatment. Hence, it is feasible that lithium, by reducing Aβ burden, promotes neurogenesis and restores cognitive functions in 3-month-old TgCRND8 mice. Moreover, the Aβ plaque-associated gliosis in the Tg mice was attenuated as the Aβ deposition was decreased by lithium, supporting an apparent anti-inflammatory role of lithium. The lithium's ability to stimulate hippocampal neurogenesis and to recover cognitive functions was lost in the 7-month-old TgCRND8 mice. The Aβ burden in the old Tg mice was mostly represented, throughout the brain parenchyma, by big and radiating plaques with a dense Aβ core which was not significantly reduced by lithium treatment, as indicated by the lack of treatment effect in the maximum Aβ plaque area. However, the lithium-treated 7-month-old group showed some radiating plaques with a ribbon-like deposit rather than a dense Aβ core and fewer small and round-shaped amyloid deposits in the main core surroundings. Furthermore, in these mice the minimum Aβ plaque area, represented by the small and round-shaped plaques, which might consist of amyloid plaques in formation, was significantly reduced by lithium treatment, once again indicating that lithium treatment prevents rather than removes/dissolves Aβ deposits. Altogether these data indicate, firstly, that lithium, while intervening only partly on previously formed plaques, represented by those with the maximum Aβ plaque area, significantly counteracts the gradual build up of Aβ deposits and, secondly, that a stronger amyloid load reduction is needed to stimulate neurogenesis and to recover cognitive functions. In 3-month-old TgCRND8 mice, the lithium-induced reduction in Aβ load was associated with a significant reduction in APP phosphorylation and in 7-month-old TgCRND8 mice with a decrease in PHF-1 tau hyperphosphorylation. It is known that lithium at therapeutic concentrations inhibits GSK-3β activity directly, by competition with the enzyme for a magnesium-binding site, and indirectly, by phosphorylation of serine-9 in the pseudosubstrate domain of the enzyme through the activation of Akt [32]. Consistent with previous findings [33], here we demonstrate that lithium treatment significantly reduced the active GSK-3β while increased the levels of inactive GSK-3β by activation of Akt. Although other mechanisms might also be involved, our data suggest that lithium, via inhibition of GSK-3β, triggers a cascade of protective events ranging from the activation of the canonical Wnt pathway to a reduction of APP phosphorylation and thereby a lesser accumulation of Aβ, to a reduction of tau hyperphosphorylation and of glia reaction in the brain of Tg mice. Likewise, recent studies have shown that therapeutic concentrations of lithium inhibit APP processing at or before the γ-secretase cleavage step and block the accumulation of Aβ peptides in brain of mice overproducing Aβ [34]. Moreover, compounds that inhibit GSK-3 could lead to the reduction of both the formation of amyloid plaques and neurofibrillary tangles. Here we demonstrate that lithium by the inhibition of GSK-3 increases β-catenin expression throughout the neocortex and hippocampus of TgCRND8 mice likely leading to the activation of Wnt/β-catenin signalling. Strongly supportive of this, our data revealed that in the lithium-treated Tg mice β-catenin is mainly expressed at the nuclear level of the newborn neurons while in the saline-treated Tg mice it remained mostly confined to the cytosol. Thus, the regulation of Wnt target genes transcription by nuclear translocation of β-catenin may underlie the increased cell proliferation and neuronal differentiation stimulated by lithium treatment. Noteworthy, Wnt signalling has been identified as an endogenous pathway that regulates hippocampal neurogenesis, as evidenced by the expression of receptors and signalling components for Wnt proteins within adult hippocampal stem/progenitor cells [10], [35], [36]. Furthermore, Wnt/β-catenin is active and triggers the expression of NeuroD1 in the hippocampal neurogenic niche of mice [35]–[37]. Inhibition of Wnt abolishes almost completely hippocampal neurogenesis and differentiation *in vivo* [38]. The enhanced neurogenesis/survival in the SGZ has been proposed as the mechanism which causes the improvement in memory test [39] and a prevention of behavioural disturbances by lithium, through an activation of Wnt signalling, has been reported in a mouse model of AD [33]. Consistent with this study we found that a 5-week lithium treatment completely recovers the water maze and step down performances of 3-month-old Tg mice with an early amyloid pathology. Concomitantly with this, the activation of Wnt/β-catenin signalling and the stimulation of neurogenesis in the SGZ were observed. In 7-month-old TgCRND8 mice, although a GSK-3β inhibition was undertaken resulting in a significant reduction in hyperphosphorylated tau protein, neither behavioural recovery nor neurogenesis activation was brought about by 5 weeks lithium treatment. The Wnt signalling dysfunctions, previously reported in these mice [25], may underlie the unfruitful effects of lithium treatment. As recently proposed for AD patients [40], it is conceivable that also in mouse models of AD, characterized by robust amyloid deposition and tau hyperphosphorylation, an extended period of lithium treatment might be necessary to counteract the disrupted Wnt/β-catenin signalling, neurogenesis and cognitive impairments. However, according to Tariot and Aisen [41] the lithium story in AD could end here and the discovery of novel agents able to attack and untie tangles represents a new hope in the therapy of AD.

In conclusion, this is the first comprehensive *in vivo* study that provides evidence that lithium, when given on time to Tg mice, significantly increased cell proliferation, neuronal differentiation, survival and maturation of newborn cells in the SGZ-GCL and significantly ameliorated behavioural performances. The lithium-induced reduction in Aβ burden may underlie its effectiveness on neurogenesis and cognitive functions. Consistent with recent studies, we demonstrated that lithium by the inhibition of GSK-3β increased β-catenin expression throughout the neocortex and hippocampus of TgCRND8 mice and thus the aforementioned results may be an outcome of activated Wnt/β-catenin signalling. Noteworthy, the lithium's ability to stimulate hippocampal neurogenesis and to recover cognitive functions declined as brain Aβ deposits increased as a function of age. Overall, our findings provide a platform for further evaluating compounds and strategies targeted at stimulating neurogenesis for treating or preventing AD in humans. As we were submitting the manuscript, the P7C3 compound capable of enhancing survival and integration of new hippocampal neurons, as well as memory, in mice and rats was discovered [42].

## Materials and Methods

### Ethics Statement

TgCRND8 and wild type mice were housed in macrolon cages with ad libitum food and water and maintained on a 12 hr light/dark cycle at a room temperature of 23°C. All experiments were carried out according to the ECC guidelines for animal care (DL 116/92, application of the European Communities Council Directive 86/609/EEC) and all efforts were made to minimize animal suffering. The protocols were approved by the National Committee for animal research, Italy (Permit number: 141/2008-B).

### Animals

Transgenic hemizygous TgCRND8 (Tg) mice and wild type (wt) control littermate male and female mice, aged 2 and 6 months, were used. Of note, in this mouse strain amyloid deposition is already detectable at 3 months of age [21], becoming robust and widespread at 7 months of age [17]. TgCRND8 mice encode a double-mutant form of amyloid precursor protein (APP) 695 (KM670/671NL+V717F) under the control of the PrP gene promoter [21].

### Lithium chloride and BrdU treatments

Two groups of TgCRND8 and wt mice, aged 2 months (Tg n = 8, wt n = 8) and 6 months (Tg n = 8, wt n = 8), were dosed with LiCl (Sigma-Aldrich, MI, Italy), or vehicle (0.9% saline). LiCl is used therapeutically at plasma concentrations between 0.2 and 1.5 milliequivalents (mEq) per liter for the treatment of bipolar disorders. TgCRND8 and wt mice received intraperitoneal (i.p.) injections of either 0.6 M LiCl (10 µl/g of body weight) or sterile saline daily for 5 weeks. This dosing paradigm was previously described by Noble et al. [43] and in our experimental condition resulted in plasma concentration of 0.223 mEq/liter 24 h after injection (n = 6), indicating that plasma lithium was maintained at physiologically relevant levels.

#### Cell proliferation and survival

To evaluate proliferation of neural progenitor cells, lithium- or saline-treated mice were administered with 5-bromo-2′-deoxyuridine (BrdU, Sigma-Aldrich, MI, Italy) (50 mg/kg of body weight) twice a day for three days and, 24 h after the last BrdU injection, mice were sacrificed (n = 5–6/group).

To assess survival of newly generated neurons, an additional group of 2-month-old wt and Tg mice treated for 5 weeks with saline or lithium, as described above, was administered with BrdU (50 mg/kg of body weight) twice a day during the last 3 days of saline or lithium treatment and sacrificed 5 weeks after the last BrdU administration (n = 4/group).

### Behavioral experiments

Tg- and wt-treated mice were behaviourally tested in the “Step-down” inhibitory avoidance test at the end of the 4th week of treatment and in the Morris water maze (MWM) test during the week 5^th^ of treatment (n = 5–6/group for 3-month-old and n = 8/group for 7-month-old mice).

#### “Step-Down” inhibitory avoidance test

The apparatus and procedures used were previously described [24]. Briefly, the inhibitory avoidance apparatus consisted of an open field grey plexiglas box (40×40 cm) with a steel rod floor. The plexiglas platform (4×4×4 cm) was set in the center of the grid floor. Intermittent electric shocks (20 mA, 50 Hz) were delivered to the grid floor by an isolated stimulator. On the first day (training test), each mouse was gently placed on the platform. When the mouse stepped down from the platform and placed all its paws on the grid floor an intermittent electric shock was delivered for 3 seconds (s). Responsiveness to the punishment in the training test was assessed by the animal's vocalization, only those mice that vocalized touching the grid with the four paws were used for the retention test in order to exclude the mice with a different pain threshold. Twenty-four hours (retention test) after training, each mouse was placed on the platform again. The latencies were measured, considering 30 s as the upper cut-off, during the training and retention tests. The tests were carried out between 10:00 A.M. and 1:00 P.M.

#### Morris Water Maze test (MWM)

The water maze apparatus consisted of a circular pool (1.2 m in diameter and 0.47 m high) made of white plastic. The pool was filled to a depth of 20 cm with water (24°–25°C) that was made opaque by the addition of non-toxic white paint. Tg and wt mice, treated with lithium or saline 0.9% were tested at week 5 of treatment in the reference memory version of MWM. We applied some modifications [44] to the method described by Chishti et al. [21] and Janus et al. [45]. Briefly, all mice underwent a reference memory training with a hidden platform (10 cm in diameter, submerged 0.5 cm under the water level), placed in the centre of one quadrant of the pool (northwest) for 4 days, with 4 trials per day, with the four starting locations varied between trials. If the platform was not located within the maximum time of 60 s, the mouse was guided to the location. The mouse was allowed 20 s on the platform. Extra-maze visual cues around the room remained in fixed positions throughout the experiment. For each trial, latency to find the platform (maximum 60 s) was recorded by a video-tracking/computer-digitizing system (HVS Image, Hampton, UK). On day 4, five hours after the last trial, the platform was removed from the pool and each mouse received one 30 s swim “probe trial”. The starting point was set in the south-east quadrant. Percentage of time spent in the target sector was recorded.

### Processing of animal tissue

At the end of behavioral tests, the mice were sacrificed by cervical dislocation and brains were rapidly removed and divided sagittally. Cortical and hippocampal samples from one hemibrain were immediately sectioned, snap-frozen and stored at −80°C in order to be processed for protein analysis. The other hemibrain was postfixed in phosphate-buffered 4% paraformaldehyde, pH 7.4, at 4°C for 48 h, then rinsed in PBS and paraffin embedded.

#### Immunohistochemistry

Immunohistochemical analyses were performed on 5 µm coronal paraffin-embedded sections prepared as previously described [24]. Briefly, sections were incubated overnight at 4°C with the primary antibodies, at the optimized working dilution, made up in PBS 0.1 M (pH 7.4) with Triton X-100 (0.3%) and BSA (5 mg/ml). On the second day, sections were incubated for 1 h with the secondary antibody at 1∶1000 dilution made up in PBS 0.1 mM plus BSA (1 mg/ml) and the immunostaining was visualized using the avidin-biotin system (Vectastain: Vector Laboratories, Burlingame, CA) and 3,3′-diaminobenzidine plus Nickel (DAB Kit: Vector Laboratories, Burlingame, CA) as the chromogen and observed by means of a light microscopy (Olympus BX40, MI, Italy). All primary antibody concentrations were titrated to provide optimal staining.

#### Fluorescent staining

Fluorescent labeling followed previously described protocols [25]. Briefly, coronal brain sections were rinsed 3 times then placed in blocking solution (PBS, pH 7.4+0.3% Triton X-100, 2g/l BSA and 5% NGS for polyclonal antibodies or 5% NHS for monoclonal antibodies), for 30 min at RT. Primary antibodies were diluted in fresh blocking solution and applied overnight at 4°C. Next, sections were washed in PBS (3×10 min) at room temperature and subsequently incubated for 2 h in the dark with the appropriate fluorescent secondary antibody (monoclonal antimouse Alexa Fluor 594 red conjugated and polyclonal anti-rabbit or anti-goat Alexa Fluor 488 green conjugated, Invitrogen, Eugene, OR) diluted 1∶200 in blocking solution. Following 3 additional rinses, sections were finally coverslipped using Vectashield water-based mounting medium with DAPI (Vector Laboratories, Burlingame, CA). The analysis of negative controls (omission of primary antibody) was simultaneously performed in order to exclude the presence of non-specific immunofluorescent staining, cross-immunostaining, or fluorescence bleed-through. All the primary antibodies used in the study are listed in Table 1.

**Table 1:** Antibodies employed in the study.

Antibody	Specific	Dilution		Host	Source
		WB	IHC		
**Aβ (1–42)**	Aβ peptide, aa 1–42	Not done	1∶200	Rabbit	Biosource
**PHF-1**	phosphorylated tau at S^396^–S^404^	1∶2000	Not done	Mouse	P. Davies
**p-GSK3α/β**	phosphorylated GSK-3 α at T^279^ and β at T^216^	1∶1000	1∶100	Rabbit	Biosource
**p-GSK3β(Ser 9)**	phosphorylated GSK-3 β at Ser 9	1∶1000	1∶100	Rabbit	Cell Signaling
**GSK 3β**	Total GSK-3 β protein	1∶1000	Not done	Rabbit	Cell Signaling
**BrdU**	Bromodeoxyuridine	Not done	1∶150	Rat	Abcam
**DCX**	Doublecortin	Not done	1∶500	Rabbit	Abcam
**NeuN**	neuronal nuclei	Not done	1∶100	Mouse	Chemicon
**β-catenin**	Total β-catenin (C-terminus)	Not done	1∶20	Goat	Santa Cruz
**GFAP**	Glial fibrillary acidic protein	1∶10000	1∶500	Rabbit	Dako
**Iba-1**	Ionized calcium binding adaptor molecule 1	Not done	1∶250	Rabbit	Wako
**p-APP(Thr668)**	Phosphorylated human APP 695 at Thr 668	1∶1000	Not done	Rabbit	Millipore
**p-Akt(Ser 473)**	Phosphorylated Akt at Ser 473	1∶1000	Not done	Rabbit	Cell Signaling
**Actin**	C-terminal actin fragment (C11)	1∶5000	Not done	Rabbit	Sigma-Aldrich

Legend: aa, amino acid; WB, western blot; IHC, immunohistochemistry.

#### Aβ plaque-load determination

To quantify Aβ plaque burden, neocortices and hippocampi of the sections stained with anti-Aβ (1–42) antibody were digitized (Olympus BX40, Olympus, Hamburg, Germany) under constant light and filter settings. Six to seven coronal brain sections, each separated by 60 µm interval, from each mouse (5–6 animals per group) were analyzed. Morphometry was conducted by using analySIS 5 software (Soft Imaging System, Münster, Germany). Colour images were converted to greyscale by extracting blue to grey values to obtain best contrast between positive immunoreactivity and background. A constant threshold was chosen for all images to detect immunoreactive staining. Plaque number, size (maximum area, minimum area) and total area were determined automatically in the parietal neocortices and hippocampus. Brain regions were based on the Paxinos and Franklin mouse brain atlas [46]. Data from the six-seven sections were summed to derive representative values for each animal for the total plaque area. Data were expressed as mean ± S.E.M.

#### Thioflavine S staining

5-µm-thick, paraffin-embedded rehydrated sections were incubated with 0.25% KMnO_4_ for 4 minutes (min), washed with water, incubated with a solution of 0.1% NaBH_4_ for 5 min and then placed in a high-concentration PO_4_ buffer (411 mM NaCl, 8.1 mM KCl, 30 mM Na_2_HPO_4_, 5.2 mM KH_2_PO_4_) pH 7.2 for 30 min. After washing Thioflavine S (Sigma-Aldrich, MI, Italy) staining was performed according to the method described by Yamamoto and Hirano [47] and the staining was observed using a fluorescence microscope (Olympus BX40, MI, Italy).

### BrdU labeling and counting

For BrdU labeling, sections were treated with 0.6% H_2_O_2_ in Tris-buffered saline (TBS; pH 7.5) to block endogenous peroxidases. DNA was denatured by exposing sections to 2N HCl for 15 min at 37°C followed by 0.1M borate buffer (pH = 8.5) for 10 min at room temperature (RT). Sections were then incubated in TBS with 0.1% Tween-20 (TBS-Tween-20), 1% BSA and 3% NHS, and then overnight at 4°C with the primary antibody anti-BrdU in the same buffer. On the second day, sections were washed (3×10 min) in TBS-Tween-20 at RT and subsequently incubated for 2 h in the dark with the appropriate fluorescent secondary antibody (polyclonal anti-rat Alexa Fluor 488 green conjugated, Invitrogen, Eugene, OR) diluted 1∶500 in blocking solution. After washes (3×10 min) with TBS–Tween-20 and briefly with water, sections were finally cover-slipped using Vectashield water-based mounting medium with DAPI (Vector Laboratories, Burlingame, CA).

An Olympus BX40 microscope coupled to analySIS∧B Imaging Software (Olympus, MI, Italy) was used to acquire representative images from the examined specimens.

Quantification of BrdU positive (BrdU^+^) cells was performed in the SGZ and in the GCL (SGZ-GCL) of the hippocampus in 6 coronal sections per mouse spaced at 80 µm. All assessments were done by experimenters who were blind to the genotype of each brain section. The counts were conducted at 40× magnification with an Olympus BX40 microscope. The corresponding surface area of SGZ-GCL sampled was measured using the analySIS∧B Imaging Software. The density of BrdU^+^ cells was calculated by dividing the number of BrdU^+^ cells by SGZ-GCL sectional area (mm^2^).

### Determination of phenotype and quantification of newborn BrdU^+^ neurons

#### Proliferation

To establish cell phenotype double-immunofluorescent labeling of BrdU with the marker for immature neurons doublecortin (DCX) or astrocytes (GFAP) was performed. Sections (6 per mouse spaced at 80 µm) pretreated for BrdU staining, were incubated with the second primary antibody in the dark overnight at 4°C in blocking solution. On the following day, sections were rinsed (3×10 min) with TBS-Tween-20 at RT and subsequently incubated for 2 h in the dark with the secondary fluorescent antibody (polyclonal anti-rabbit Alexa Fluor 594 red conjugated, Invitrogen, Eugene, OR) diluted 1∶500 in blocking solution. After washes (3×10 min) with TBS-Tween-20 and briefly with water, sections were finally cover-slipped using Vectashield water-based mounting medium with DAPI (Vector Laboratories, Burlingame, CA).

Quantification of new born immature neurons was calculated as the percentages of BrdU^+^ cells expressing DCX in SGZ-GCL. The GFAP marker was used to exclude a glial phenotype of new born cells.

#### Cell Survival

To evaluate the survival of newborn cells triple-immunofluorescent labeling of BrdU with markers for mature neurons (NeuN) and for astrocytes (GFAP) was performed. Sections (6 per mouse spaced at 80 µm) after BrdU staining were incubated with NeuN in the dark overnight at 4°C in blocking solution. The next day, sections were rinsed (3×10 min) with TBS-Tween-20 at RT and incubated for 2 h in the dark with the fluorescent secondary antibody (anti-mouse Alexa Fluor 594 red conjugated, Invitrogen, Eugene, OR) diluted 1∶500 in blocking solution. After washes (3×10 min) with TBS-Tween-20, sections were incubated with GFAP in the dark overnight at 4°C in blocking solution. The third day, sections were rinsed (3×10 min) with TBS-Tween-20 at RT and incubated for 2 h in the dark with the fluorescent secondary antibody (anti-rabbit Alexa Fluor 350 blue conjugated, Invitrogen, Eugene, OR) diluted 1∶500 in blocking solution. Subsequently sections were rinsed (3×10 min) with TBS-Tween-20 and briefly with water and finally cover-slipped using Vectashield water-based mounting medium.

The density of newborn mature neurons was calculated by dividing the number of BrdU^+^ cells expressing NeuN by SGZ-GCL sectional area (mm^2^).

In every study the analysis of negative controls (omission of primary antibody) was simultaneously performed in order to exclude the presence of non-specific immunofluorescent staining, cross-immunostaining, or fluorescence bleed-through.

### Western blotting analysis

Tissue samples were homogenized in ice-cold lysis buffer containing 2 mM NaPP, 4 mM PNFF, 1 mM Na_3_VO_4_, 1mM PMSF, 20 µg/ml leupeptin, 30 µl/ml aprotinin. Equal amounts of protein samples (40 µg) were applied to SDS-polyacrylamide gels and subjected to electrophoresis as previously described [25]. Bands were normalized to β-actin level to provide a control for equal loading.

### Data Analysis

The MWM training data and phospho-GSK-3β(Ser9) and phospho-Akt levels were analyzed by two-way-ANOVA followed by Bonferroni's post comparison test. One-way ANOVA, followed by Bonferroni's post-hoc test, was used to analyse the density of BrdU^+^ cells. Student's t-test was used to analyse Aβ plaque burden and PHF-1, phospho-GSK3β(Tyr216), phospho-APP(Thr668) levels. Statistical analyses were carried out with GraphPad Prism 4 and statistical significance was defined as P<0.05. Data are reported as mean values ± standard error of the mean (S.E.M).
